# Case Report: Biological treatment of epidermolysis bullosa acquisita: report on four cases and literature review

**DOI:** 10.3389/fimmu.2023.1214011

**Published:** 2023-07-12

**Authors:** Konrad Szymański, Cezary Kowalewski, Ewelina Pietrzyk, Katarzyna Woźniak

**Affiliations:** Department of Immunodermatology, Medical University of Warsaw, Warsaw, Poland

**Keywords:** epidermolysis bullosa acquisita, EBA, rituximab, infliximab, IVIG, inflammatory bowel diseases

## Abstract

Epidermolysis bullosa acquisita (EBA) is a chronic, recurrent autoimmune subepidermal bullous disease characterized by the presence of autoantibodies targeting type VII collagen -- basement membrane zone antigen. Standard therapy for EBA includes a combination of systemic corticosteroids and dapsone; however, severe cases may require advanced treatment. The current article reports on four EBA cases in which biologics: infliximab, rituximab (Rtx), and intravenous immunoglobulin (IVIG) were applied. All patients fulfilled the clinical and immunological criteria of EBA: they presented tense blisters healing with atrophic scars on the skin on traumatized areas and in mucous membranes. The diagnosis of EBA was established using numerous techniques: direct and indirect immunofluorescence, salt split skin, ELISA, Fluorescence Overlay Antigen Mapping using Laser Scanning Confocal Microscopy. Since all the patients did not achieve long-term remission on standard treatment (prednisone, dapsone) due to ineffectiveness or side effects of drugs, they eventually were treated with biologics leading to extraordinary skin improvement and stopping the disease for 1-3 years. Biologics in all patients were tolerated very well. No side effects were observed during application as well as multi-month follow-up. The presented cases provide a premise that biological drugs can be a valuable component of EBA therapy.

## Introduction

Epidermolysis bullosa acquisita (EBA) is a chronic autoimmune subepidermal blistering disease. It is characterized by the presence of autoantibodies targeting type VII collagen, a major component of anchoring fibrils between the dermal-epidermal junction in the basement membrane (BMZ) (1). It usually occurs in adulthood (median onset age 50) (2), although pediatric cases were also reported (3–5).

Two main clinical EBA types can be distinguished as mechanobullous and inflammatory EBA. The mechanobullous variant features skin fragility and blister formation in the trauma-prone body regions. Skin lesions in the inflammatory EBA type occur in both traumatic and non-traumatic areas. In addition to skin, different mucous membranes can also be affected. Blisters and subsequent erosions within oral and nasal cavities, throat, and anogenital areas are further complicated by dysphagia, dysphonia, dysuria, and dyspareunia. Patients suffer from pain and itchy skin. This chronic disease is characterized by recurrent blistering, which may gradually lead to skin scarring with milia, atrophy of skin appendages, or dental problems. Severe cases result in blindness, esophageal stricture, hand joint contracture, or degenerative changes in distal phalanges. These clinical EBA characteristics result from complex pathology consisting of circulating antibodies directed to type VII collagen and the autoreactive T cells (6). This makes EBA therapy constantly challenging.

The most applied systemic treatment features a combination of corticosteroids and dapsone. In addition, colchicine, azathioprine, or mycophenolate mofetil were also reported to provide good adjuvant treatment to prednisone (7). Despite the availability of various treatment schemes, EBA treatment based on classical immunosuppressive drugs is often ineffective and causes side effects. Attempts have therefore been made to treat the most severe EBA cases with new biological agents used in other dermatoses.

This paper reports on four EBA cases who were successfully treated with biological drugs: infliximab, rituximab and/or intravenous immunoglobulin (IVIG).

## Materials and methods

### Patients

The study included four patients, two females and two males, who met clinical and immunological EBA criteria. All four patients had a long clinical history of tense blisters, atrophic scars and milia on traumatized skin areas, affected mucous membranes, and nail dystrophy (detailed characteristics shown in **Table 1**). All four patients were positive in direct immunofluorescence test (DIF), with the presence of at least one type of linear deposits of IgG, IgA, or C3 along the BMZ. Only in two patients (Cases 3 and 4), it was possible to characterize the target antigen (type VII collagen) with ELISA. The other two patients tested negative in serum studies using IIF on salt-split skin, ELISA and BIOCHIP (immunological characteristics of the patients shown in **Table 1**).

**Table 1:** Clinical and immunological characteristics of EBA cases.

No.	Sex, age (in years) at onset and at biological treatment	Previous treatments	Comorbidities	Biological treatment	Follow up/ Maintenance therapy	DIF	IIF, ELISA, FOAM-LSCM
1	F 64 73	Tetracycline Prednisone+Dapsone Clobetasol propionate	Hypertension Glaucoma T2DM Osteoporosis Vasculitis Pulmonary embolism	Rituximab 2×1 g	15 months Dapsone (50 mg/day)	deposits of linear IgG (forming u-serrated pattern), IgA and C3 along the BMZ in intact skin, and on the roof and the floor of an adjacent spontaneous blister	IIF on SS: negative ELISA: negative FOAM-LSCM: ND
2	F 43 49	Prednisone+Dapsone Colchicine Tetracycline Mycophenolate mofetil Sulfasalazine	No	IVIG 6×140 g	30 months Dapsone (50 mg/day)	interrupted linear deposits of IgG along the BMZ	IIF on SS: negative ELISA: negative FOAM-LSCM: ND
3	M 21 25	Methylprednisone+Dapsone Mycophenolate mofetil	Intellectual disability	IVIG 8×180 g Rituximab 2×1 g	19 months Methylprednisolone 4 mg/day (Good response only between IVIG infusions Follow up since Rtx infusion)	linear deposits of IgG and C3 along the BMZ on the floor of the natural skin blister	IIF on SS: circulating IgG on the floor of the blister ELISA: reaction of circulating IgG antibodies with collagen type VII FOAM-LSCM: IgG deposits located below collagen type IV
4	M 19 23	Mesalazine+Azathioprine+Methylprednisone/ Prednisone	Ladd’s bands Crohn’s disease	Infliximab 12×350 mg	12 months Mesalazine 4 g/day+Azathioprine 100 mg/day	linear IgG deposits (forming u-serrated pattern) and IgA along the BMZ	IIF on SS: negative ELISA: reaction of circulating IgG antibodies with collagen type VII FOAM-LSCM: ND

F, female; M, male; DIF, direct immunofluorescence of skin biopsy; ELISA, enzyme-linked immunosorbent assay with 6 antigens (Dsg1, Dsg3, envoplakin, collagen type VII, NC16a-BP180kD, 230kD); FOAM-LSCM, fluorescence overlay antigen mapping using laser scanning confocal microscopy; IIF, indirect immunofluorescence; IVIG, intravenous immunoglobulins; ND, not done; Rtx, rituximab; SS, salt split skin substrate; T2DM, type 2 diabetes.

Informed consent was obtained from all subjects involved in the study. The study was conducted according to the guidelines of the Declaration of Helsinki and approved by the Institutional Ethics Committee of the Medical University of Warsaw.

## Case reports and results

Clinical and laboratory features of the Cases discussed are presented in **Table 1**.

### Case 1

**Figure 1:**
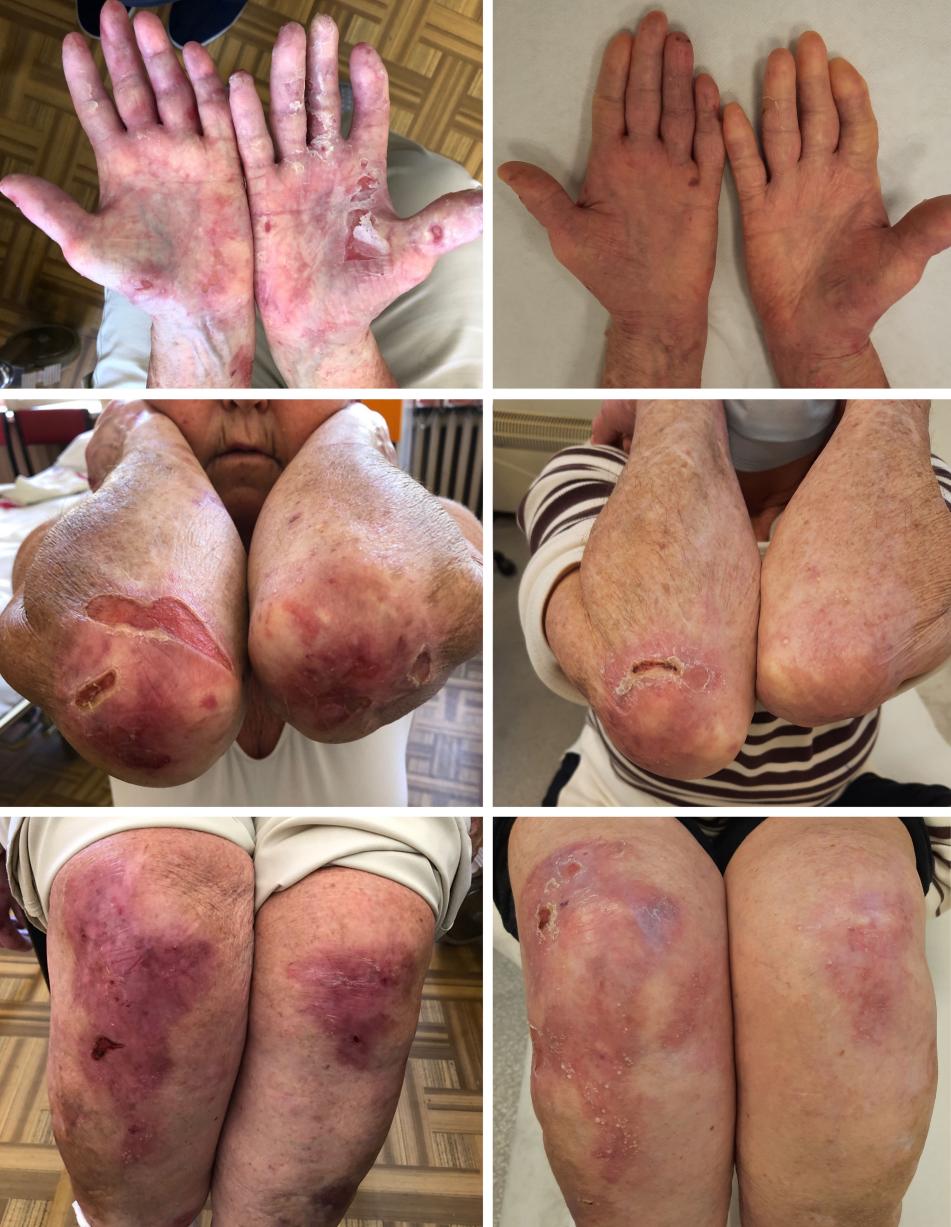
Clinical manifestation of EBA in Case 1 before (left) and after treatment (right).

This female patient was diagnosed with EBA at the age of 66 years, following a two-year history of vesicullobullous eruptions localized symmetrically on crura. The patient was initially diagnosed with bullous pemphigoid (BP) and treated with tetracycline. Following a correct diagnosis, the therapy was augmented to a combination of prednisone (40-20 mg/day), dapsone (50-100 mg/day) and ultrapotent topical corticosteroids (clobetasol propionate). Nevertheless, periods of partial remission and exacerbations resulted in diffuse atrophic scars with milia localized symmetrically on her elbows, knees, buttocks, hands, and feet and complete nail loss on eight fingernails. She also occasionally presented erosions in the oral cavity. Long-term systemic steroid therapy led to severe complications in the patient, such as hypertension, glaucoma, type 2 diabetes, osteoporosis, and pulmonary embolism. At the age of 72, the patient was twice infused with 1g of rituximab during treatment with 20 mg of prednisone daily. This treatment continuously led to the complete healing of all erosions (**Figure 1**). During 12-month follow-up, the patient reported significantly less skin fragility and eruption of only a few blisters caused by the patient’s increased physical activity. Additionally, the patient experienced much faster healing of erosions when compared to the time before rituximab therapy (several days, as opposed to several weeks). The patient is currently being administered only dapsone with a 50 mg/day dosage as maintenance therapy.

### Case 2

**Figure 2:**
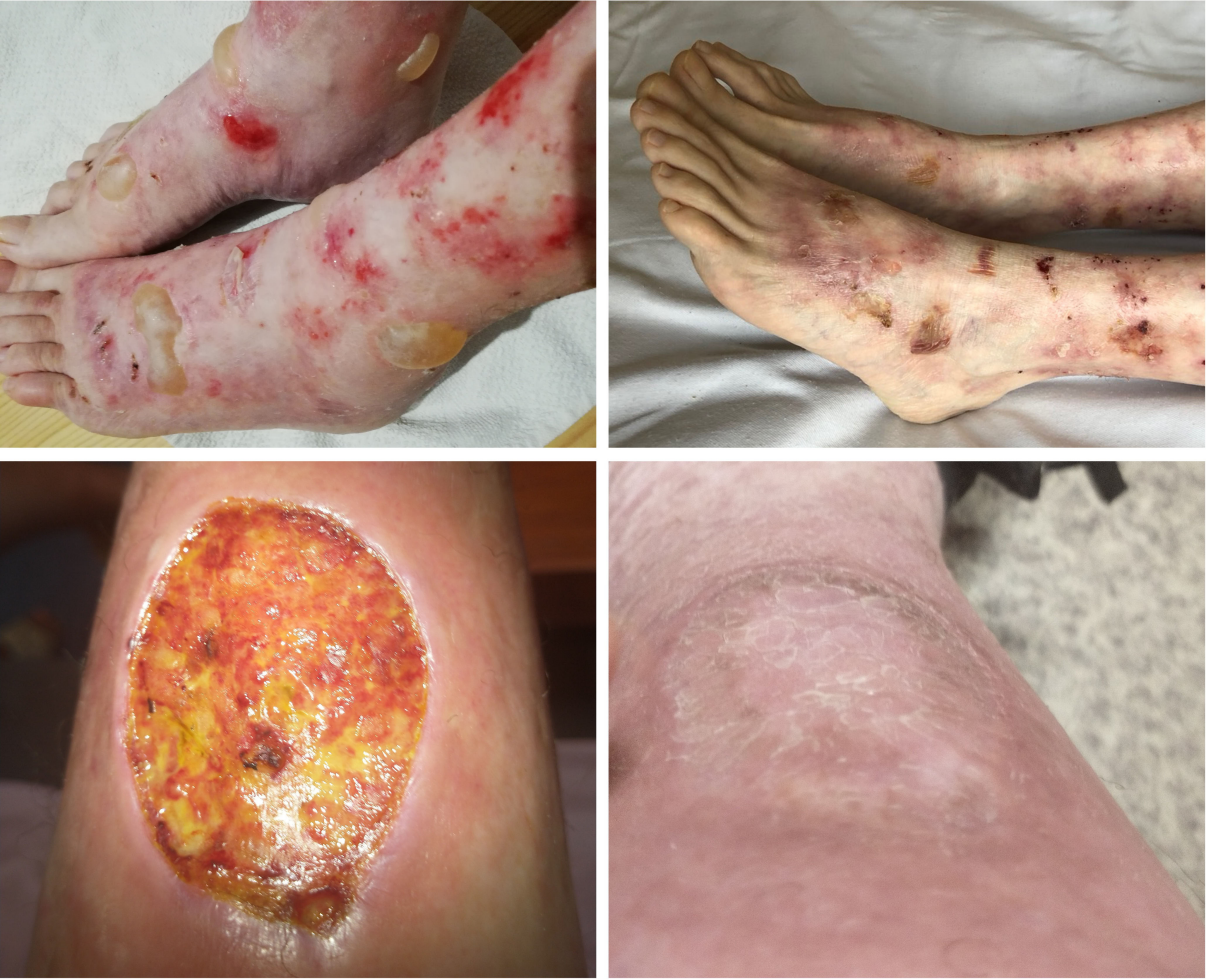
Clinical manifestation of EBA in Case 2 before (top left) and after treatment (top right) and Case 3 before (bottom left) and after treatment (bottom right). Figures of Case 3 (bottom) show a wound caused by trauma - before treatment with rituximab, post-traumatic wounds healed significantly longer than after the treatment.

The patient was a 53-year-old healthy female with a 10-year history of EBA. The patient’s EBA was characterized by well-tense large blisters, symmetrically located on the dorsal aspects of hands, feet, forearms, elbows, knees, shins, and abdomen, which were healing with atrophic scars. Occasionally, she developed erosions in the oral cavity. Other mucous membranes and nails were not affected. The dominant therapy featured the combination of prednisone and dapsone in doses adjusted to the severity of the disease. Other treatments, i.e., colchicine, tetracycline, mycophenolate mofetil, and sulfasalazine, failed to control the EBA course or were not well-tolerated by the patient. Consequently, therapy with intravenous immunoglobulins (IVIG) was initiated in a total of six pulses of IVIG (2 g/1 kg of body weight) at irregular intervals (4-12 weeks) due to restrictions related to the COVID-19 epidemic. A gradual reduction in the severity of the disease was observed over the next 12 months. New blisters appeared with decreased frequency and healed more easily. Despite the lack of complete clinical remission, a significant improvement was observed during the follow-up period, lasting 46 months (**Figure 2**).

### Case 3

The patient was a 29-year-old male who has slight intellectual disability, and developed EBA at the age of 21. He presented blisters and erosions mainly located on traumatized areas (hands, knees, feet); however occasionally, he had disseminated blisters on the trunk. In periods of skin exacerbations, the patient also developed oral erosions. Nails were slightly affected. Initially, methylprednisolone in combination with dapsone was used, which, however, did not lead to remission of the disease but resulted in the formation of extensive stretch marks. Further treatment involved a six-month course of mycophenolate mofetil (3 g/d), which was also unsuccessful. Finally, the patient received 4 pulses of IVIG (2g/kg b.w.) at 4-6 weeks intervals, which reduced disease activity for 12 months. Due to EBA re-exacerbation, IVIG therapy was repeated (4 pulses of IVIG at a dose of 2g/kg b.w. at intervals of 4-8 weeks), reducing the number and frequency of blistering. However, persistent improvement was not observed. Consequently, two infusions of rituximab in a dose of 1g each were administered at a two-week interval leading to the gradual healing of existing erosions and stopping the eruption of new blisters. The patient has been in clinical remission for 2 years taking methylprednisone at a maintenance dose of 4 mg/d (**Figure 2**).

### Case 4

**Figure 3:**
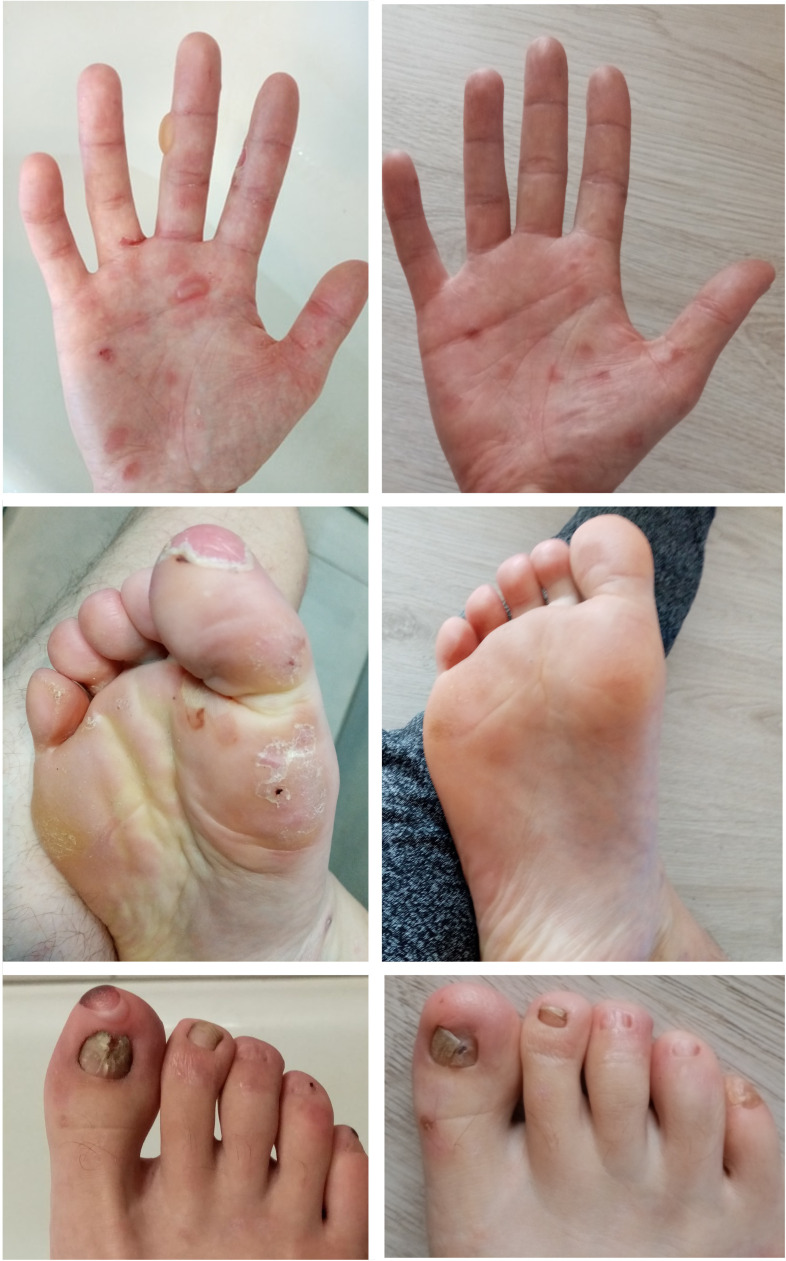
Clinical manifestation of EBA in Case 4 before (left) and after treatment (right).

The patient was a 25-year-old male suffering from Ladd’s bands. The patient underwent laparotomies due to intestinal obstructions in childhood and a periodically active cutaneous fistula on the abdomen. The patient was diagnosed with Crohn’s disease at the age of 18. A year after the diagnosis, well-tense blisters healing with atrophic scars began to appear symmetrically on the dorsal aspects of the hands. These gradually spread to forearms, arms, and then to feet, shins, and knees. Ulcers were also present in the oral cavity and esophagus. In addition, dystrophy occurred in most toenail plates (with fingernails preserved). EBA was diagnosed based on clinical symptoms and laboratory tests. Direct immunofluorescence test revealed linear deposits of IgG and IgA within the BMZ (**Supplementary Figure 1**). ELISA test revealed the reaction of circulating IgG antibodies with collagen type VII. Initially, the patient’s treatment entailed the combination of mesalazine, azathioprine, and methylprednisolone, with periodic prednisone usage. This treatment, however, did not effectively control either EBA or Crohn’s disease. In addition, the patient began to show signs of malnutrition. An extensive inflammatory process was also observed in the entire gastrointestinal tract, together with the progression of cutaneous lesions manifesting as scarring and the loss of toenails. When the patient was 23 years old, therapy with infliximab was initiated. A total of 12 pulses were administered at 350 mg doses at 8-week intervals. During the biological therapy, a remission of both diseases was achieved. At present, 19 months following the last infliximab infusion, a colonoscopy confirmed remission of Crohn’s disease. No new blister eruptions on the skin were observed. The patient reported experiencing skin fragility, although to a significantly lesser extent. Skin wounds were healing at a faster rate compared to the patient’s condition before biological therapy (**Figure 3**). Treatment with mesalazine and azathioprine is maintained.

## Discussion

In general, the first line of EBA therapy is a combination of prednisone and dapsone. However, the application of other adjuvants, such as methotrexate, colchicine, cyclophosphamide, or cyclosporine, has also been reported. Most EBA cases are isolated autoimmune disease however, EBA may coexist with many different disorders. Recently, it has been found that 55 different diseases had significantly different prevalences among EBA patients compared to the general population. Of these, 23 diseases were identified as risk factors for EBA (8). In these cases, alternative therapy may be required, as exemplified by our patients.

### Infliximab in EBA treatment

From the cases analyzed here, Case 4 proves an interesting point for discussion from a pathological viewpoint. This patient developed EBA within one year of Crohn’s disease diagnosis, which is consistent with other reports (9). It is likely that because of the ongoing intestinal inflammation, collagen VII epitopes, typically invisible to the immune system, are exposed and modified. In genetically susceptible cases, antibodies against such neoantigens may be formed in the gut, binding collagen type VII epitopes in the skin. This could lead to the formation of subepidermal blisters (10, 11).

Therefore, biologicals by reducing inflammation in the gut may play a role in treating EBA, as we observed in our patient after infliximab therapy. Infliximab is a monoclonal antibody directed against TNF-α that reduces infiltration, adhesion and chemotaxis of inflammatory cells as well as enables reduction in proinflammatory IL-1 and IL-6 cytokines levels concurrently leading to IBD recovery and long-lasting clinical EBA remission. A similar observation was made by Vicente et al. (2008) (9), who presented a 22-year-old female with EBA and 2-year-long history of CD who, after treatment with infliximab in combination with conventional immunosuppressive agents, gained remission of EBA.

Noteworthy is the lack of literature reports on treating isolated EBA with infliximab, although recent reports justify the use of anti-TNF drugs in isolated EBA. In research on a mice model of EBA, an inhibition of TNF function with etanercept or a monoclonal antibody against mouse TNF resulted in significantly less severe disease progression and a reduction in the number of macrophages in skin lesions (12).

Since cytokine dysregulation seems to play an important role in the pathogenesis of EBA, other anti-inflammatory drugs might be effective for both isolated and IBD-related cases of EBA. In a recent report of a case of EBA associated with CD, ustekinumab was applied (13). The authors described a case of 36-year-old female with a long history of recurrent EBA and 1 year-long history of CD who was resistant to oral corticosteroids. However, biological therapy with ustekinumab finally led to the total remission of EBA. Ustekinumab is an IgG1 human monoclonal antibody that binds with high specificity to the p40 protein subunit shared by the cytokines IL-12 and IL-23, therefore, suppresses the Th1 and Th17. As authors emphasize that EBA response did not correlate with CD response to ustekinumab, which could be in contradiction to the gut neoantigens theory. It is possible that observed rapid EBA improvement may be a result of the ustekinumab-dependent modulation of the immune response against the Th1 pathway rather than the elimination of the source of neoantigens; however, it does not exclude the possibility that long-term remission of EBA may be the result of gut healing.

Although IBD has long been recognized as a risk factor for EBA (2, 14–17), recent research on medical records of 1344 EBA cases has challenged this association. The authors did not identify inflammatory bowel diseases as risk factors or sequelae of EBA (8).

Regardless of the pathogenetic mechanisms, the elimination of enteritis seems to be a pivotal one of the therapeutic approaches in IBD-related EBA. Biologicals should therefore be considered the first line therapy and promptly initiated to avoid severe, irreversible complications of EBA (esophagus stricture, nail dystrophy) in those cases.

The other three Cases in the current report represent mechanobullous EBA without a history of other autoimmunological diseases. Here, the initial classical treatment was ineffective and caused side effects. Based on the literature, only IVIG and rituximab treatment would achieve complete remission in refractory EBA like in the presented Cases. So far, both therapies have been recognized the most in the treatment of pemphigus (18, 19).

### IVIG in EBA treatment

The IVIG action mechanism in treating autoimmune diseases is still a matter of debate. Two main pathways are distinguished as follows: (a) through the interaction of the Fcγ and F(ab)2 regions (the receptor action); and (b) by their effects on a cellular level (the non-receptor action) (20). Recently, research based on the mouse model of EBA showed that IVIG reduced the amount of anti-type VII collagen antibodies in the skin and sera (21). This, in turn, is indicative of an FcRn-dependent mode of action. In a nonreceptor-mediated fashion, IVIG showed antioxidative properties by scavenging extracellular ROS (20). Moreover, IVIG were able to impair complement activation and served as a substrate for proteases – key events in EBA pathogenesis (22).

Two of four of our Cases were treated with IVIG. Case 2 achieved significant reduction of disease severity after IVIG therapy without side effects. This observation is supported by the reports of 29 EBA cases treated with IVIG summarized by Koga et al. (23). Twenty-six of these cases achieved at least partial remission, and only three patients did not benefit from IVIG infusions (13–31). In Case 2, IVIG infusions were found to be sufficient treatment, while in Case 3, additional treatment with rituximab was necessary. Such combined treatment has led to the complete healing of skin erosions and remission lasting to this day. This confirms that combined therapy with rituximab and IVIG may be more beneficial than monotherapy in exceptional cases. Other authors (24) also demonstrated that a combination of rituximab with another therapy, such as immunoabsorption, is more effective than both of these treatments in monotherapy.

### Rituximab in EBA treatment

The most severe in our group, Case 1 was initially treated conventionally with prednisone and dapsone, however, she rapidly developed significant side effects, and the treatment had to be changed. After infusion of rituximab (Rtx), this patient eventually achieved complete healing of skin and oral erosions and remained in good general condition for one year.

In the literature, we found 21 cases of EBA treated with rituximab (24–36) summarized by Koga et al. (23). Rituximab selectively binds to the CD20 transepithelial antigen present on the surface of normal and neoplastic B lymphocytes, leading to the reduction of antibodies production and, consequently, to the healing of skin lesions. Sixteen patients from the literature showed clinical improvement during the follow-up period; four showed no response to treatment, and one died from pneumonia 2 weeks after Rtx administration. In the patient group that benefited from the treatment, eleven patients could discontinue corticosteroids, and seven experienced a total remission of EBA in the follow-up lasting from 6 to 60 months. Differences in the duration of remission after rituximab treatment are likely to be associated with the mode of action of rituximab, similar to pemphigus. In general, reduction in B-lymphocyte titre has a temporal nature, and it is usually observed for up to 6 months. The B-lymphocyte population is restored within 10-18 months since pro-B cells do not express CD20 antigen.

A review of the literature identified the three most common rituximab infusion protocols: (a) low-dose rheumatologic (LDRA) – 2×0.5 g in a 2-week interval; (b) high-dose rheumatologic (HDRA) – 2×1 g in a 2-week interval; and (c) lymphoma schema – 375 mg/m^2^ per infusion (37). Two of our cases (Case 1 and Case 3) were twice treated with rituximab at the dose of 1 g with a 2-week interval, which led to remission lasting 1 and 2 years, respectively. It is noteworthy that rituximab was tolerated well by both our patients and patients reported in the reviewed papers. Complications associated with rituximab in patients with pemphigoid-group diseases were observed in 39% of patients. These were mainly infections (44–46). Interestingly, Wu et al. reported on an exceptional adverse event of EBA being provoked by rituximab. Here, a 54-year-old female was treated with Rtx due to the idiopathic thrombocytopenic purpura relapse. The day after the Rtx infusion, pruritic blisters on both arms and chest were observed. DIF showed linear deposition of IgG and C3 at the BMZ, and IIF on salt-split skin revealed exclusive dermal binding of circulating IgG antibodies, which confirmed EBA. Skin lesions regressed after induction treatment with intravenous methylprednisolone followed by prednisone. After six weeks, EBA relapsed, but the treatment with a low dose of prednisone combined with topical steroid therapy was sufficient to clear the erosions in 5 days. Wu et al. concluded that this case might have represented a delayed type of hypersensitivity reaction to rituximab (35). It is, however, possible that the performed antibodies had already been present in the cell membranes of lymphocytes, and the rituximab infusion caused their release.

## Conclusion

It is difficult to compare the effectiveness of rituximab and other biologicals in EBA across studies. This is due to the difference in dosing regimens and clinical subtypes and the varying severity and duration of the disease. Still, according to our clinical experience and literature data in severe EBA cases with rapid progress, biologicals should be considered promptly. This is both to avoid complications, such as finger contractures or narrowing of the esophagus, as well as to improve patients’ quality of life. Biologicals are increasingly being applied within the EBA treatment. This will provide further evidence, which will help to establish the most accurate EBA management methods.

## Data availability statement

The original contributions presented in the study are included in the article/**Supplementary Material**. Further inquiries can be directed to the corresponding author.

## Ethics statement

The studies involving human participants were reviewed and approved by Institutional Ethics Committee of Medical University of Warsaw. The patients/participants provided their written informed consent to participate in this study. Written informed consent was obtained from the individual(s) for the publication of any potentially identifiable images or data included in this article.

## Author contributions

All authors cared for the patients and contributed to the writing of this report. All authors contributed to the article and approved the submitted version.

## Supplementary material

Supplementary Figure 1 U-serrated pattern in direct immunofluorescence test in Case 4.
