# Supplementary figures and images for: Case Report: Biological treatment of epidermolysis bullosa acquisita: report on four cases and literature review
Source: Front Immunol. 2023 Jul 12;14:1214011. doi: 10.3389/fimmu.2023.1214011 (PMC10371012; doi:10.3389/fimmu.2023.1214011)

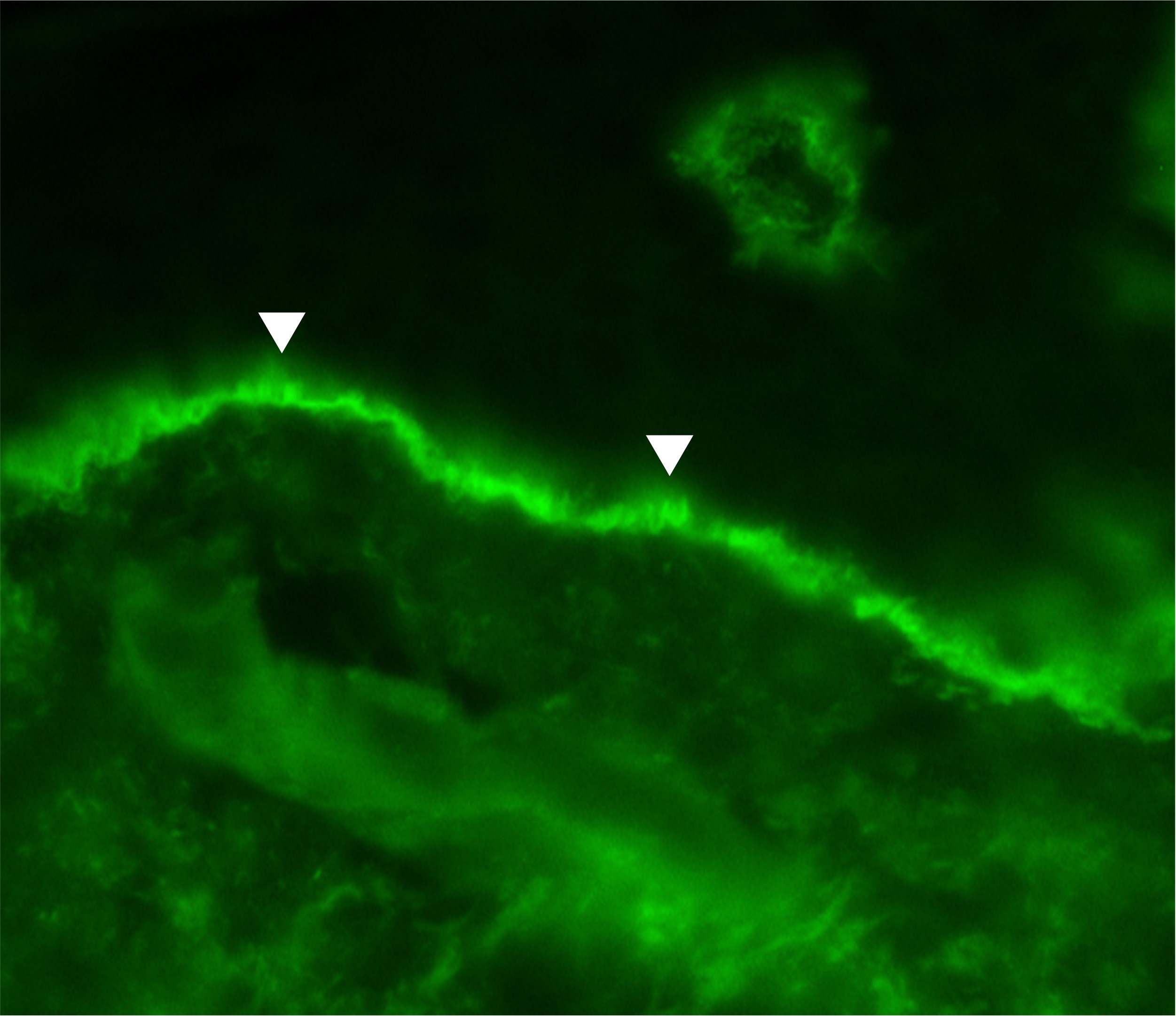

Supplement: Supplementary Figure 1 — U-serrated pattern in direct immunofluorescence test in Case 4. [file Image_1.jpeg]
